# Pentoxifylline as an adjunct therapy in children with cerebral malaria

**DOI:** 10.1186/1475-2875-9-368

**Published:** 2010-12-21

**Authors:** Bertrand Lell, Carsten Köhler, Betty Wamola, Christopher HO Olola, Esther Kivaya, Gilbert Kokwaro, David Wypij, Sadik Mithwani, Terrie E Taylor, Peter G Kremsner, Charles RJC Newton

**Affiliations:** 1 KEMRI Centre for Geographic Medicine Research (Coast), Kilifi, Kenya; 2 Medical Research Unit, Albert Schweitzer Hospital, Lambaréné, Gabon; 3 Institute of Tropical Medicine, University of Tübingen, Tübingen, Germany; 4 Department of Pharmaceutics & Pharmacy Practice, School of Pharmacy, University of Nairobi, Nairobi, Kenya; 5 Department of Biostatistics, Harvard School of Public Health, Boston, Massachusetts, USA; 6 Department of Pediatrics, Harvard Medical School, Boston, Massachusetts, USA; 7 Department of Cardiology, Children's Hospital Boston, Massachusetts, USA; 8 College of Osteopathic Medicine, Michigan State University, East Lansing, Michigan, USA; 9 Blantyre Malaria Project, University of Malawi College of Medicine, Blantyre, Malawi; 10 Institute of Child Health, University College London, London, UK; 11 London School of Hygiene and Tropical Medicine, London, UK

## Abstract

**Background:**

Pentoxifylline (PTX) affects many processes that may contribute to the pathogenesis of severe malaria and it has been shown to reduce the duration of coma in children with cerebral malaria. This pilot study was performed to assess pharmacokinetics, safety and efficacy of PTX in African children with cerebral malaria.

**Methods:**

Ten children admitted to the high dependency unit of the Kilifi District Hospital in Kenya with cerebral malaria (Blantyre coma score of 2 or less) received quinine plus a continuous infusion of 10 mg/kg/24 hours PTX for 72 hours. Five children were recruited as controls and received normal saline instead of PTX. Plasma samples were taken for PTX and tumour necrosis factor (TNF) levels. Blantyre Coma Score, parasitemia, hematology and vital signs were assessed 4 hourly.

**Results:**

One child (20%) in the control group died, compared to four children (40%) in the PTX group. This difference was not significant (p = 0.60). Laboratory parameters and clinical data were comparable between groups. TNF levels were lower in children receiving PTX.

**Conclusions:**

The small sample size does not permit definitive conclusions, but the mortality rate was unexpectedly high in the PTX group.

## Background

The mortality of severe falciparum malaria in children admitted to African hospitals has not decreased significantly in recent decades despite the introduction of new anti-malarial drugs, including the rapidly acting artemisinins. Disruption of the pathogenic mechanisms may improve the outcome.

The pathogenesis of severe malaria is unclear and several different processes may be responsible. Enhanced production of the cytokine tumour necrosis factor (TNF), induction of nitric oxide, cytoadherence and sequestration, rosetting (the aggregation of parasitized and non-parasitized red blood cells), and decreased red blood cell (RBC) deformability are thought to contribute to the pathogenesis of severe malaria [1,2].

Pentoxifylline (PTX), a methylxanthine, which acts as a phosphodiesterase inhibitor, has effects on many of these processes. It was initially developed to increase RBC deformability and enhance blood flow in the microvasculature. It leads to an overall improvement in haemorheological characteristics such as RBC deformability, blood viscosity, platelet aggregation, and plasma fibrinogen concentration [3]. It may be particularly useful in malaria, since it disrupts rosettes [4], prevents neuronal cell damage [5], and reduces TNF levels [6-9]. In premature neonates with sepsis, which may share pathogenetic processes with severe malaria, continuous infusion of PTX decreases the mortality rate [10].

Pentoxifylline as an adjunctive therapy for severe malaria was first proposed in the early 1990 s and to date four clinical trials have investigated the efficacy of the drug in severe malaria. Trials in adults have shown conflicting results; a trial from India showed a significant reduction in duration of coma and mortality [11], but trials from Thailand [12] and Germany [13] showed no effect. A study of Burundi children with cerebral malaria also showed a significant reduction in the duration of coma [9], but the sample size was small and an optimal dose was not established.

The pharmacokinetic properties of PTX are well described in healthy adults [3], but little is known about pharmacokinetics in African children with severe malaria. The present study therefore set out to characterize the pharmacokinetik porperties of PTX and to measure its effect on pathophysiologic parameters, with the aim to determine a dose to be tested in large multi-centre trials.

## Methods

### Study area and study population

The study was approved by the Institutional Review Boards at the Michigan State University, USA, the International Center for Infectious Diseases Research protocol review committee within the National Institute of Allergy and Infectious Diseases, U.S. National Institutes of Health, USA and the National Ethics Committee of Kenya. The trial was registered at ClinicalTrials.gov, with the identifier NCT00133393.

The study was conducted between January 2002 and September 2004, in the coastal region of Kenya, an area of moderate to high malaria transmission [14]. Recruitment was temporarily stopped for 18 months in 2002/2003 due to patient safety concerns after the first death. All children admitted at the Kilifi District Hospital were eligible if they fulfilled the following inclusion criteria: between nine months and eight years of age; cerebral malaria (defined as peripheral parasitaemia with asexual forms of *P. falciparum*; inability to localize a painful stimulus (evaluated 30 minutes after correcting hypoglycaemia (blood glucose < 2.2 mmol/l) in patients who present with hypoglycaemia and 30 minutes after cessation of convulsive activity in patients who are convulsing on admission); and informed consent given by parents or guardians. Exclusion criteria were: mean blood pressure (BP) < 60 mmHg (mean BP = 2/3 diastolic BP + 1/3 systolic BP); platelet count < 50/nL; spontaneous bleeding; haematocrit < 20% or haematocrit between 20-25% with parasitaemia > 10%; neck stiffness; or clinical evidence of lower respiratory infections.

### Study design

The study was planned as a step-wise dose-escalation controlled study of ten subjects in each group, starting with 10 mg/kg, increasing by 10 mg/kg with 40 mg/kg as the maximum dose. The first four patients were assigned to the control group and two controls were to be added between each dosage group. However, after preliminary analyses, the study was stopped after 15 patients due to patient safety concerns raised by the National Ethics Committee of Kenya and the Data and Safety and Monitoring Board (DSMB).

### Study procedures

Children were admitted to the high-dependency research ward of the Kilifi District Hospital and were treated with a loading dose of 15 mg/kg quinine and a maintenance dosage of 10 mg/kg quinine every 8 hours thereafter [15]. Chloramphenicol and benzylpenicillin were administered to all children to cover the possibility of bacterial meningitis until the cultures were available. Seizures lasting longer than 5 minutes were treated with paraldehyde, phenytoin, or phenobarbital [16]. Mannitol was infused when evidence of increased intracranial pressure was observed, mainly retinal vein engorgement or papilledema.

Out of the 15 children recruited, 10 children received a continuous infusion of 10 mg/kg/24 hours PTX in 0.9% saline (5 mg PTX/mL) for 72 hours. Five children (the first four and the last one recruited) acted as controls and received continuous infusion of normal saline only.

On admission, demographic details of the patient, history of disease, and clinical findings (vital signs, oxygen saturation, respiratory status, Blantyre coma score) [17] were recorded. Laboratory on admission included parasitaemia, full blood count and differential white cell count, glucose, blood culture, blood gas analysis, electrolytes, creatinine, lactate, and aspartate transaminase (AST). Parasite density was counted per 100 white blood cells or, for high parasitaemia, per 500 red blood cells and expressed in parasites per μL.

During the study, parasitaemia, glucose, and haematocrit were assessed 4-hourly until three consecutive slides were negative for parasites. Vital signs were assessed quarter-hourly during the first 4 hours and 4-hourly thereafter. Blantyre coma score was assessed every 4 hours. A full blood count and differential was performed at 12, 24, 48, and 72 hours. Lactate was measured at 4, 8, 12, and 24 hours. Creatinine and AST were measured after 72 hours. Blood for determination of TNF, PTX and metabolite (1-(5'-hydroxyhexyl)-3,7-dimethylxanthine) concentrations was taken on admission and at 1, 2, 4, 8, 12, 24, 48, and 72 hours after PTX administration. Blood was collected in heparinized tubes, centrifuged to separate plasma, and the plasma was stored frozen (-20°C) until assayed to PTX and its major metabolite.

Follow-up visits were conducted at one and three months after admission. During these visits, neurological status was assessed and the examinations performed on admission were repeated.

### Laboratory methods

Plasma PTX and metabolite concentrations were determined using a validated High Performance Liquid Chromatographic method developed in our laboratory. Peak drug/metabolite concentrations and the corresponding times were the experimentally observed values, while the area under the plasma drug/metabolite concentration-time curve was determined using the trapezoidal method [18]. However, there were insufficient data to allow for determination of post-infusion elimination kinetics.

TNF levels were measured from plasma samples by standard sandwich enzyme-linked immunosorbent assay using pairs of cytokine specific monoclonal antibodies according to the manufacturer's instructions (Flexia, Biosource International, Nivelles, Belgium) with a detection limit of 11 pg/mL. Each plate included a standard curve of recombinant human cytokine. All specimens were measured in duplicate and the mean value was used in all analyses. The optical density of each well was measured at 450 nm by a dual-wavelength plate reader (Mikrowin, Orthvath, Germany). The correlation between optical densities ranging from 1 to 1000 pg/ml was strong in all cases.

### Statistical analysis

Outcome variables included the number of children who died, the number who survived with neurological sequelae (inability to sit or stand or walk, pick up objects, see, hear and/or speak), and resolution time of coma, parasitaemia, and fever. Parasite clearance time was defined as the time (i.e., hours after start of pentoxifylline) when the first of two consecutive 4-hourly peripheral blood films was negative. Coma resolution time was defined as the time since start of PTX when a Blantyre Coma Score of 5 was first noted. Fever clearance time was defined as the time since start of PTX when the first of five consecutive 4-hourly rectal temperatures was below 37.5°C. Laboratory values were compared between intervention groups by flagging values outside of normal ranges. Serious adverse events were defined as any untoward medical occurrence that is life-threatening, results in death, or in prolongation of hospitalization. Fisher's exact tests were used to compare categorical variables and Wilcoxon rank sum tests and Student's t-tests were used to compare continuous variables.

## Results

Admission characteristics of the children allocated to the PTX and control groups were similar (Table 1). With the exception of rectal temperature (p = 0.03), there were no statistically significant differences among study groups, at a significance limit of 0.05.

**Table 1:** Clinical and laboratory parameters on admission

	PTX (N = 10)	Controls (N = 5)
Sex (Male)	3 (30%)	3 (60%)
Age (years)	3.6 ± 1.1	3.6 ± 1.9
Duration of illness (days)	2 (1 - 5)	2 (1 - 3)
Seizures before admission	8 (80%)	4 (80%)
Blantyre Coma Score	2 (0 - 2)	2 (0 - 3)
Adelaide Coma Score	6 (3 - 9)	7 (4 - 7)
Rectal temperature (°C)	38.9 ± 1.1	37.6 ± 0.8
Mean blood pressure (mm Hg)	74 ± 15	74 ± 11
Presence of respiratory distress	2 (20%)	1 (20%)
Parasitaemia (/μL)	40,991 (2,331 - 380,000)	29,988 (1,950 - 264,000)
Haemoglobin (g/dL)	8.2 ± 1.4	9.7 ± 1.4
Glucose (μmol/L)	7.0 ± 3.9	5.5 ± 3.8
Creatinine (μmol/L)	65 ± 27	68 ± 33
Platelets (/nL)	77 (23 - 432)	354 (69 - 730)
pH	7.341 ± 0.124	7.347 ± 0.114
Base excess (mmol/L)	-4.4 (-18.2 - 13.3)	-7.2 (-14.6 - 2.2)

Data are reported as N (%), mean ± standard deviation, or median (range)

Outcome variables are shown in Table 2. Five children died; 4 in the PTX group and 1 in the control group, resulting in an overall mortality rate of 33%. Although the mortality rate was high in the PTX group, it was not statistically different from that of the control group. Respiratory arrest was the cause of all deaths and signs of raised intracranial pressure were present in each case [19]. There were no significant differences between study groups for any outcome variable; in particular, coma resolution time and number of children with seizures. Fever resolution time and parasite clearance time were higher in the PTX group, but the differences were not statistically significant.

**Table 2:** Outcome variables by treatment assignment

	PTX	Control	p
All subjects	(N = 10)	(N = 5)	
Serious adverse events	2 (20%)	0 (0%)	0.52
Seizures during stay	4 (40%)	4 (80%)	0.28
Deaths	4 (40%)	1 (20%)	0.60
Time to death (hours)	35 (8 - 54)	10	-
Among survivors	(N = 6)	(N = 4)	
Parasite clearance time (hours)	43 ± 9	31 ± 8	0.05
Fever resolution time (hours)	29 ± 17	17 ± 10	0.28
Coma resolution time (hours)*	8 (4 - 36)	8 (4 - 12)	0.69
Duration of hospitalization (days)	3 (3 - 17)	4 (3 - 15)	0.52
Neurological impairment after 72 hours	1 (17%)	2 (50%)	0.50
Neurological sequelae at 3 months	0 (0%)	1 (25%)	0.40

* Restricted to subjects with no neurologic impairment at 72 hours

Data are reported as N (%), mean ± standard deviation, or median (range)

Two children experienced a serious adverse event and the PTX infusion was stopped as a consequence. One child developed hypotension (mean blood pressure below 60 mmHg), and the other demonstrated frequent vomiting (seven episodes over a four-hour time period) and gastrointestinal bleeding. The latter patient had *Salmonella *sepsis on admission, required two blood transfusions and was subsequently found to be HIV positive. Two children in the PTX group needed transfusion, compared to none in the control group. The blood culture was positive in two subjects in the PTX group (one *Salmonella *and one *Staphylococcus epidermidis*), and no positives were found in the control group.

Twelve children (80%) had haemoglobin below 8 g/dL on at least one occasion, 2 (13%) had aspartate transaminase above 150 U/L at least once, and 3 (20%) had creatinine above 100 μmol/L at least once. There was no statistically significant difference in the number of children with laboratory values outside of the normal range between the control and intervention groups for any laboratory parameter.

**Figure 1:**
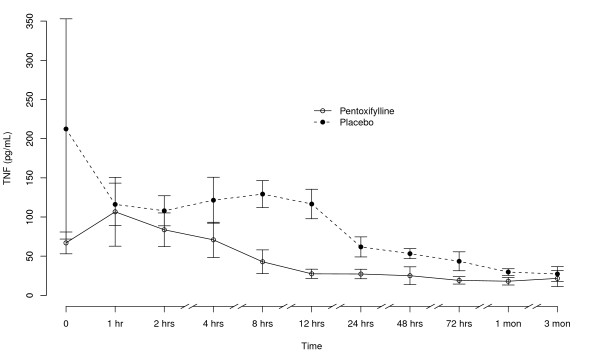
**Tumour necrosis factor levels over time by treatment assignment**. Values are plotted as mean ± 95% confidence intervals.

TNF values were consistently and significantly reduced in the PTX group starting 2-4 hours after start of administration (Figure 1). There was a difference between the study groups at baseline, but not at hour 1. The pharmacokinetic properties are summarized in Table 3. There was considerable variation in the PTX and metabolite pharmacokinetic parameters.

**Table 3:** Pharmacokinetic parameters of pentoxifylline and its metabolite

	PTX (N = 10)	PTX metabolite (N = 9)
C_max _(ng/mL)	151 (82 - 332)	94 (35 - 291)
T_max _(hours)	10 (2 - 24)	12 (4 - 24)
AUC_0-24 _(ng ^. ^h/L) *	2828 (1743 - 3052)	1567 (831 - 1585)
AUC_0-∞ _(ng ^. ^h/L) *	7641 (5123 - 9177)	2314 (852 - 2855)

* Data restricted to seven subjects with sufficient data points

Data are median (range)

C_max _: maximum concentration_; _T_max _: time to maximum concentration;

AUC_0-24 _: area under the curve (0 to 24 hours); AUC_0-∞ _: area under the curve (0 to infinity)

## Discussion

Pentoxifylline has often been proposed as an adjunct therapy in cerebral malaria [20-22]. This is based on the evidence that it reduces mortality in sepsis [10], a disease that seems to share similar pathogenetic features with severe malaria.

A study conducted in 56 Burundian children showed that PTX (10 mg/kg/day) caused a significant reduction in median coma resolution time, which was six hours in the PTX group compared to 46 hours in the control group. The reduction in mortality (17% vs. 0%) was not statistically significant, but the sample size may have been too small to detect such a difference. However this was not a double blind randomized trial, and it is unclear if the dose used was the optimal dose.

In adults, the studies of PTX in cerebral malaria are contradictory. In a study of 52 Indian adults with cerebral malaria, there was a significant reduction in duration of coma, but not mortality in the PTX group [11]. In a study of 45 adult Thais with severe malaria (18 had cerebral malaria), no differences were seen when PTX was used at higher doses (20 mg/kg daily and 40 mg/kg daily) [12]. A study performed in Germany in a heterogeneous cohort of malaria, which included three cerebral malaria cases, showed no effect of PTX (20 mg/kg daily) [13].

The rationale for the present study was the need to describe pharmacokinetics in children with cerebral malaria in order to decide on a dosage for large-scale studies. However, we found that the efficacy of the drug was associated with more side effects than the Burundian study, despite using the same dose. The study was not designed as an efficacy study and as a consequence there was no randomization of the intervention and the number of subjects was small, due to the early termination of the study. The difference between the Burundian and this study remains unexplained, since the entry criteria and regimens appear to be similar.

The study shows that PTX was effective in reducing TNF levels, even at low PTX dosages. In the neonatal sepsis, PTX was given at a dosage of 30 mg/kg per day, which led to a reduction of TNF and other cytokines compared to placebo [10]. In Thai adults, a high dose (PTX infusion of 40 mg/kg daily) significantly decreased TNF and IL-6 levels compared to a lower dose (20 mg/kg daily) with significantly lower TNF-receptor plasma concentrations after 12 and 24 hour of high-dose PTX infusion, but there were no differences between the groups with regard to IL-6 receptor levels [8].

The pharmacokinetic properties of PTX administered orally are well described in adults [3]. Considerably less information is available on the pharmacokinetics of the drug administered as an infusion and in children, sick or healthy. Due to the short elimination half-life of PTX and its metabolite, a continuous infusion is probably required when used in cerebral malaria. The basic pharmacokinetic parameters measured in the present study show a considerable variation compared to plasma levels seen during continuous infusion in healthy adults [23,24]. All children had a severe systemic disease and some had co-morbid conditions, such as bacteraemia. PTX undergoes a high first-pass metabolism and liver impairment is known to profoundly alter the pharmacokinetics of PTX. However, for a drug with a high hepatic extraction ratio, decreased hepatocellular activity would be expected to have only a minor effect on clearance following intravenous infusion. Thus, the observed wide variability in pharmacokinetic parameters in this study partly reflects the limited number of subjects for whom complete drug concentration-time profiles were available for analysis.

## Conclusions

The use of PTX in children with cerebral malaria remains unresolved. It reduces the TNF levels, but whether this effect reduces mortality can only be examined by further studies in moderate malaria and a large-scale multi-center trial.

## Competing interests

The authors declare that they have no competing interests.

## Authors' contributions

BL: participated in the data and sample collection, data analysis and drafted the manuscript. CK: participated in the data and sample collection. BW: participated in the data and sample collection. CHOO: carried out the data management and participated in the data analysis. EK: participated in the data and sample collection. GK: carried out the analysis and interpretation of pharmacokinetic data. DW: participated in the design of the study, the data management and data analysis. SM: participated in the design of the study, and in the data and sample collection. TET: participated in the design of the study, and the writing of the study protocol. PGK: participated in the design of the study, and the writing of the study protocol. CRJCN: participated in the design of the study, the writing of the study protocol and carried out the study coordination. All authors read and approved the final manuscript.
